# A Bee Trp-Arg Dense Peptide with Antiproliferation Efficacy against the Prostate Cancer Cell Line DU145

**DOI:** 10.3390/cimb46030144

**Published:** 2024-03-10

**Authors:** Ye-eun Kim, Ki-Young Kim

**Affiliations:** 1 Graduate School of Biotechnology, Kyung Hee University, Seocheon, Giheung, Yongin 17104, Republic of Korea; antyeeun@khu.ac.kr; 2 Department of Genetics and Biotechnology, Kyung Hee University, Yongin 17104, Republic of Korea

**Keywords:** bee Trp-Arg dense peptide, prostate cancer, androgen-independent prostate cancer

## Abstract

Prostate cancer accounts for 14% of male cancer-related fatalities in the UK. Given the challenges associated with hormone-based therapies in the context of androgen-independent prostate cancer, there is an imperative need for research into anticancer drugs. N0821, a peptide belonging to the Trp-Arg dense region and derived from the homologous region of various bee species, shows substantial potential for an anticancer effect. Both MTT assays and 3D spheroid assays were conducted to substantiate its antiproliferation potential and strongly indicated the antiproliferation effect of N0820 (WWWWRWWRKI) and N0821 (YWWWWRWWRKI). Notably, the mechanism underlying this effect is related to the downregulation of CCNA2 and the upregulation of CCNE1. Cell cycle arrest results from the reduction of CCNA2 in the S/G2 phase, leading to the accumulation of CCNE1. Our peptides were predicted to make an α-helix structure. This can act as an ion channel in the cell membrane. Therefore, we analyzed genes implicated in the influx of calcium ions into the mitochondria. Trp-Arg dense-region peptides are known for their antibacterial properties in targeting cell membranes, making the development of resistance less likely. Hence, further research in this area is essential and promising.

## 1. Introduction

Prostate cancer stands as a leading cause of mortality among men and ranks among the most prevalent malignancies, predominantly affecting individuals aged 50 years and older [1]. The progression of prostate cancer is typically slow and often attributed to the overproduction of steroid hormones.

Currently, prostate cancer management involves therapeutic modalities such as radiation therapy, surgical intervention, and chemotherapy [2,3]. A commonly used primary approach to managing prostate cancer involves surgical or pharmacological methods, such as androgen deprivation therapy. Although androgen deprivation therapy works well in the early phases of the condition, cancer subsequently advances to an androgen-resistant stage, for which no established efficacious therapy is currently available [4]. Consequently, there is an imperative need for research aimed at developing anticancer pharmaceutical agents capable of addressing androgen-independent prostate cancer.

The DU145 cell line, lacking androgen receptor expression, is frequently used as a model to evaluate the effectiveness of anticancer compounds against androgen-independent prostate cancer [5]. Peptides containing Trp-Arg-rich motifs have been recognized for their antimicrobial [6] and anticancer properties [7]. Notable examples include tritrpticin [1,8,9], lactoferricin [10,11], and indolicidin [12].

Among various types of bees, *Apis mellifera* is commonly utilized in honeybee research. Proline- and arginine-rich peptides, such as Apidaecin derived from the lymph fluid of *Apis mellifera*, are well-known antimicrobial peptides [13]. Additionally, melittin, extracted from the venom of *Apis mellifera*, is an alkaline polypeptide composed of 26 amino acid residues. Melittin selectively inhibits Ras-overexpressing cancer cells through a mechanism involving phospholipase A2 hyperactivation, calcium influx, and subsequent destruction of the transformed cells [14].

We have identified a peptide sequence resembling tritrpticin in the DNA sequences of multiple bee species. Tritrpticin, a widely known antimicrobial peptide derived from pig bone marrow, exhibits one of the shortest sequences among antimicrobial peptides, making it easily developable for treatment. A variant of tritrpticin with an arginine bound to its C-terminal specifically targets bacterial cell membranes, demonstrating antibacterial effects [15].

Numerous anticancer drugs must enter the inside of cells for their therapeutic action [16]. However, peptides with target cell membranes usually show less risk of developing resistance [17,18]. This characteristic may contribute to the resistance prevention capabilities of these peptides [19].

## 2. Materials and Methods

### 2.1. Cell Line and Cell Culture

The DU145 prostate cancer cell line was obtained from the Korean Cell Line Bank and cultured in Roswell Park Memorial Institute 1640 (RPMI 1640) medium supplemented with 10% Fetal Bovine Serum (FBS) and 1% penicillin/streptomycin (p/s). The cells were incubated in a humidified incubator at 37 °C with 5% CO_2_.

### 2.2. Identification and Structural Analysis of Trp-Arg Peptides Derived from Honey Bee DNA

We identified Trp-Arg-enriched motifs within the peptides of honeybees, conducted comparative analyses of these shared sequences across diverse bee species, and subsequently synthesized the resulting peptide with the help of the BIOSTEM company (2193, Seobu-ro, Jangan-gu, Suwon-si, Gyeonggi-do, the Republic of Korea). The specific sequence employed is detailed in Table 1. Descriptions detailing the characteristics of each bee species are provided in Table 2 [20,21,22,23]. The peptide structures were predicted using PEP-FOLD4 “https://bioserv.rpbs.univ-paris-diderot.fr/services/PEP-FOLD4/ (accessed on 29 December 2023)” [24] (Table 3).

### 2.3. MTT Assay

The DU145 cells were seeded in a 96-well microplate at a density of 5 × 10^3^ cells per well and incubated at 37 °C with 5% CO_2_ for 24 h. Following this, the cells were treated with the indicated concentrations of the Trp-Arg dense-region peptide (0, 0.5, 1, 5, 10, and 20 μM) in a serum-free medium for an additional 24 h.

Subsequently, the cells were treated with a solution containing 0.5 mg/mL of 3-(4,5-dimethylthiazol-2-yl)-2,5-diphenyltetrazolium bromide (MTT) and incubated at 37 °C for 3 h [25]. Formazan crystals were dissolved by adding 100 μL of dimethyl sulfoxide (DMSO) to each well. The resulting mixture was gently agitated for 15 min in a dark room, and absorbance was then measured using a microplate reader (BioTek Instruments, Sejong, the Republic of Korea) at a wavelength of 540 nm [26].

### 2.4. The Formation of Prostate Cancer Spheroids and Three-Dimensional (3D) Culture Assay

The DU145 cells were seeded at a density of 5 × 10^3^ cells per well in a 96-well SPL3D™ Cell Floater (SPL, #34896) and incubated at 37 °C with 5% CO_2_ for 24 h. Subsequently, the culture medium was treated with 20 μM of the Trp-Arg dense-region peptide to evaluate its impact on spheroid formation capacity [27,28]. After 3 and 4 days, images of the spheroids were captured using a microscope equipped with a camera (EVOS^®^ FL Cell Imaging System, Thermo Fisher Scientfic, Waltham, MA, USA), and their areas were quantified using Image J software V 1.8.0 [29].

### 2.5. Flow Cytometry

The DU145 cells were initially seeded at a density of 5 × 10^4^ cells per well in a 6-well plate and cultured under standard conditions at 37 °C with 5% CO_2_ for 24 h. Subsequently, the Trp-Arg dense-region peptide was added to serum-free medium at concentrations of 0, 10, and 20 μM, and the cells were further cultured for an additional 24 h. After sample preparation, the specimens were stained with Annexin V and propidium iodide (PI) [30] following the instructions provided by the BD Pharmingen^TM^ FITC Annexin V Apoptosis Detection Kit (#556547) [31].

### 2.6. Quantitative Real-Time PCR

Following the cell preparation, consistent with previous experiments, total RNA was extracted using TRIzol reagent (Life Technology, Thermo Fisher Scientific, Waltham, MA, USA) in accordance with the manufacturer’s protocol. Subsequently, cDNA was synthesized using 1 μg of the total RNA [32]. Quantitative real-time PCR was conducted using Q2X Sybr Green qPCR Master Mix (CellSafe, Yongin, Republic of Korea) [33]. The primer sequences used are detailed in Table 4, with GAPDH as the reference gene for normalization [34]. The gene expression level was calculated as fold change values and represented as delta-delta Cq, a widely used method for relative gene expression quantification.

### 2.7. Western Blot

Following the cell preparation, the same as in previous experiments, the proteins were isolated, and a BCA assay was conducted to determine the protein concentration. A total of 30 μg of protein lysates was loaded into each well and separated using sodium dodecyl sulfate–polyacrylamide gel electrophoresis (SDS-PAGE). Subsequently, the proteins were transferred onto a polyvinylidene difluoride (PVDF) membrane, and blocking was performed using a 5% BSA solution dissolved in TBST buffer (20 mM Tris-HCl, 150 mM NaCl, and 0.1% Tween 20, pH 7.6) [25]. Primary antibodies, including p-p53 (sc-51690), GAPDH (sc-2577), JNK (sc-7345), p-JNK (sc-6254) p38 (sc-535), p-p38 (sc-17852-R) (Santa Cruz Biotechnology, Santa Cruz, CA, USA), and p53 (9282s) (Cell Signaling Technology, Beverly, MA, USA), were incubated overnight at 4 °C. Afterward, a secondary antibody was applied for 1 h, and the protein bands were visualized using an enhanced chemiluminescence (ECL) reagent (Bio-Rad, Hercules, CA, USA) and detected using UVITEC imaging system equipment (UVITEC, Cambridge, UK). The Western blot bands were quantified using ImageJ software V 1.8.0 [37].

### 2.8. JC-1 Staining

After 24 h of peptide treatment, the cells were prepared using trypsin EDTA. Subsequently, JC-1 staining was conducted in accordance with the manufacturer’s protocol, and quantification was performed using the FlowJo_v10.9.0 software [38].

### 2.9. Statistical Analysis

The data are presented as the mean ± standard deviation (SD). Each experiment was conducted in duplicate and repeated a minimum of three times for statistical robustness. Significance was assessed using a one-way analysis of variance (ANOVA), with statistical significance indicated as follows: * for *p* < 0.05, ** for *p* < 0.01, and *** for *p* < 0.001. GraphPad Prism software version 5.0 was used to perform all the statistical analyses [39].

## 3. Results

### 3.1. N0820 and N0821 Had Antiproliferation Activity against Androgen-Independent Prostate Cancer Cells

**Figure 1:**
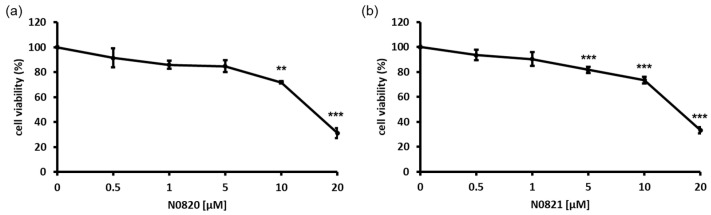
Bee Trp-Arg dense peptides, N0820 and N0821, had antiproliferation effect on DU145 cell line in 2D environment. (**a**) indicates cytotoxic effects of N0820 peptide on prostate cancer, and (**b**) indicates cytotoxic effects of N0821 peptide on prostate cancer. Data are expressed as mean ± standard deviation (SD). Each experiment was conducted in duplicate and repeated a minimum of three times. Significance was determined using one-way analysis of variance (ANOVA) with significance levels denoted as ** *p* < 0.01, *** *p* < 0.001.

An MTT assay was performed to assess the growth inhibition of the bee-derived Trp-Arg dense peptides N0820 and N0821 against DU145, an androgen-independent prostate cancer cell line. The DU145 cells were exposed to the indicated concentrations of each peptide (0, 0.5, 1, 5, 10, and 20 μM) for 24 h. As shown in Figure 1, each peptide dose-dependently inhibited the growth of the DU145 cells by 74% and 68% with a treatment of 20 μM, respectively. These findings indicate the antiproliferation effect of both peptides on DU145 cell proliferation.

### 3.2. N0820 and N0821 Suppressed the Prostate Cancer Spheroid Size in a 3D Environment

**Figure 2:**
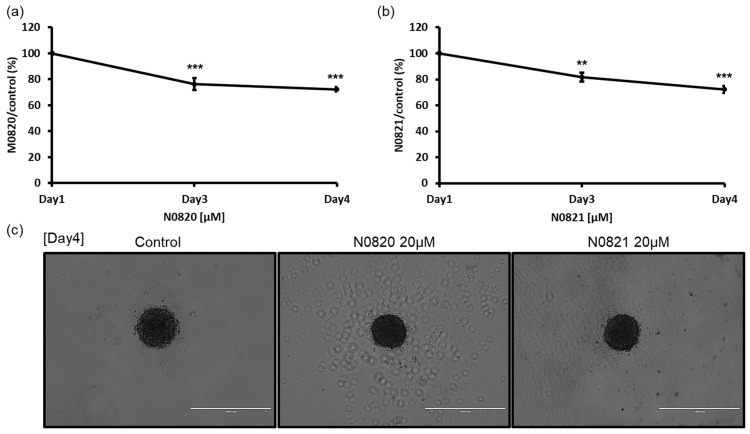
Bee Trp-Arg dense peptide inhibited the spheroid growth of DU145 cell. On the 3rd and 4th days after exposure to 20 μM of N0820 and N0821 conducted both a visual analysis (**c**) and a quantitative assessment of the tumor spheroid dimensions. (**a**,**b**) indicate the quantification was performed using Image J v 1.8.0 and it revealed a noticeable reduction in size, which exhibited a direct correlation with the concentration of the peptide. Significance was determined using one-way analysis of variance (ANOVA) with significance levels denoted as ** *p* < 0.01, *** *p* < 0.001.

To verify the potential antiproliferation activity of the Trp-Arg dense-region peptides, N0820 and N0821, within a 3D microenvironment, the cells were cultured using the SPL3D™ Cell Floater with or without the peptides. Following treatment with 20 μM of each peptide on day 3 and day 4, a notable reduction in spheroid formation was observed. In comparison to the control group treated with water, both peptide-treated groups exhibited only 74% of spheroids at day 4 (Figure 2). These results suggest the substantial inhibitory impact of these peptides on spheroid formation in a 3D culture setting.

### 3.3. N0820 and N0821 Had Ion-Channel-Like Activity and Overloaded Ca^2+^ into the Mitochondria by Reducing the Expression and Phosphorylation of p53

The Trp-Arg dense peptide potentially exhibits ion-channel-like characteristics, with its three Trp residues functioning as selective conduits for positively charged ions. This peptide can integrate into the lipid bilayer in a manner reminiscent of an ion channel, leading to an influx of Ca^2+^ ions [40].

**Figure 3:**
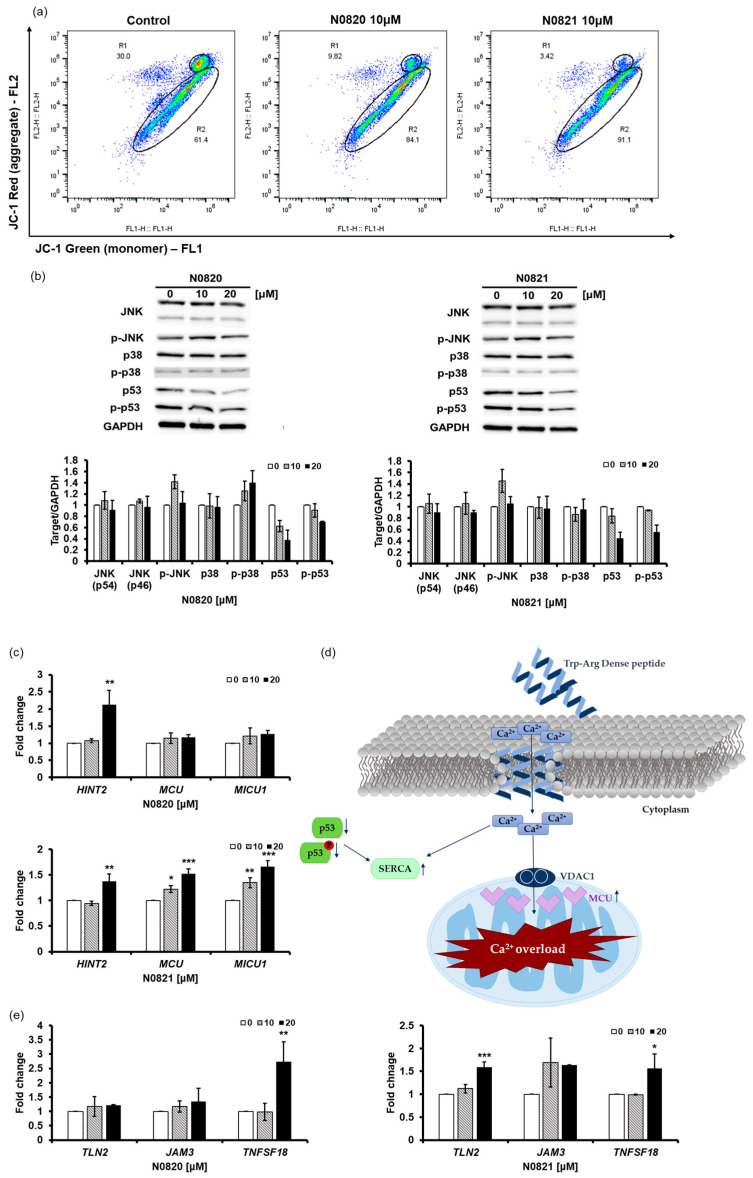
N0820 and N0821 inhibits mitochondrial function via p53-Ca^2+^ signaling. (**a**) After peptide treatment, JC-1 staining was employed to identify mitochondrial activity. (**b**) The expression of p53, p-p53, and other proteins was analyzed via Western blot. (**d**) An illustrative schematic of the inhibition of mitochondrial activity via the p53 and Ca^2+^ signaling pathways. Trp-Arg dense peptide makes a channel in the cell membrane, resembling a Ca^2+^ channel, leading to an influx of Ca^2+^ into the cytoplasm. This influx of Ca^2+^ results in a decrease in p53 and p-p53, which induces the expression of SERCA and MCU, causing Ca^2+^ overload in the mitochondria. (**c**) MCU increases due to decrease of p53, which causes MICU to increase. Moreover, there is notable augmentation in HINT2 levels, a factor intricately associated with MCU. (**e**) The changes in genes occurred in the process where HINT2 regulates the mitochondrial Ca^2+^ uniporter (MCU) complex to induce mitochondrial Ca^2+^ influx. The overload of Ca^2+^ triggers cell apoptosis. Significance was determined using one-way analysis of variance (ANOVA) with significance levels denoted as * *p* < 0.05, ** *p* < 0.01, *** *p* < 0.001.

The JC-1 staining results revealed a notable decrease in aggregated JC-1 fluorescence in the cells treated with N0820 (20.18% reduction) and N0821 (26.58% reduction) compared to the control cells (Figure 3a). And the Western blot results demonstrate that the treatment of N0820 and N0821 triggers the inhibition of p53 and p-p53 expression and not much change in any other protein (Figure 3b). The reduction in the p53 and p-p53 levels results in the upregulation of Sarco/endoplasmic reticulum Ca^2+^ ATPase (SERCA) and mitochondrial Ca^2+^ uniporter (MCU) expression [41]. It can be ascertained that the influx of Ca^2+^ into the cytoplasm subsequently enters the mitochondria, owing to the heightened expression of SERCA and MCU caused by decreased p53 and p-p53 expression. The influx of Ca^2+^ assists in inhibiting mitochondrial activity.

Our investigation revealed substantial upregulation of the expression levels of HINT2 and MCU (Figure 3c). Notably, HINT2 demonstrates the capacity to induce apoptosis by modulating the mitochondrial calcium influx through the regulation of the mitochondrial calcium uniporter (MCU) complex [42]. This elevation in HINT2 and MCU expression was accompanied by a consequential overload of Ca^2+^ within the mitochondria. Moreover, our findings indicated a concurrent increase in the expression of MICU1, a pivotal regulator of mitochondrial Ca^2+^ homeostasis. This upregulation of MICU1 expression corresponds to its role in modulating and fine-tuning the mitochondrial Ca^2+^ uptake, thus signifying a feedback mechanism in response to the heightened activity of HINT2 and MCU.

### 3.4. N0820 and N0821 Induced Apoptosis in the Androgen-Independent Prostate Cancer Cell Line DU145 

**Figure 4:**
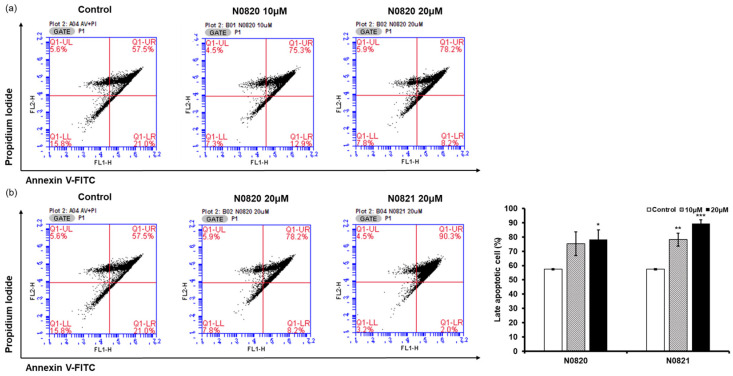
N0820 and N0821 induced prostate cancer cell apoptosis. (**a**) Apoptosis was verified by performing Annexin V and PI staining on cells 24 h after treatment with N0820. (**b**) Apoptosis was verified by performing Annexin V and PI staining on cells 24 h after treatment with N0821. The stained cells were subsequently analyzed using flow cytometry. Significance was determined using one-way analysis of variance (ANOVA) with significance levels denoted as * *p* < 0.05, ** *p* < 0.01, *** *p* < 0.001.

Flow cytometry analysis was conducted to determine whether the peptide’s antiproliferation effect was associated with the induction of apoptosis in the prostate cancer cells. When examining the P1 cell population, it was observed that in the cells treated with N0820, late apoptosis increased by 17.8% at 10 μM and 20.7% at 20 μM compared to the control group (Figure 4). Similarly, for cells treated with N0821, late apoptosis was enhanced by 25.5% at 10 μM and 32.8% at 20 μM relative to the control. These results suggest that N0820 and N0821 peptides are capable of inducing apoptosis.

### 3.5. N0820 and N0821 Induced S/G2 Phase Cell Cycle Arrest in the Androgen-Independent Prostate Cancer Cell Line DU145

**Figure 5:**
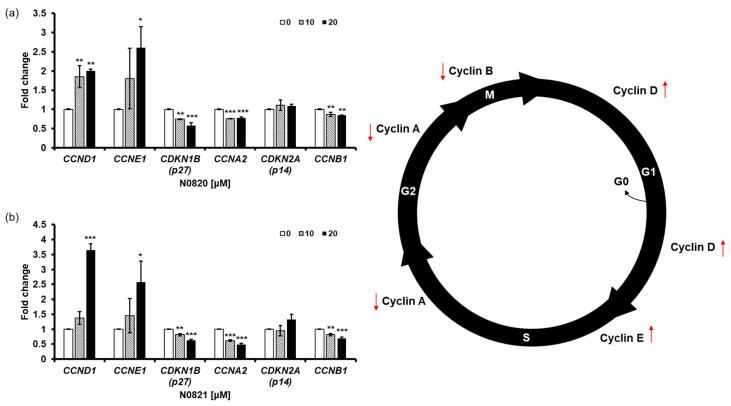
N0820 and N0821 induced S/G2 phase cell cycle arrest. (**a**,**b**) evaluate the expression level of genes related to the cell cycle using qRT-PCR after treating N0820 and N0821 peptides to prostate cancer cell lines. Significance was determined using one-way analysis of variance (ANOVA) with significance levels denoted as (* *p* < 0.05, ** *p* < 0.01, *** *p* < 0.001).

To determine the impact of the peptides N0820 and N0821 on cell cycle regulation, we conducted qRT-PCR to assess the expression of genes related to the cell cycle. We observed an increase in the relative mRNA expression of CCNE1 and CCND1, while there was a decrease in the relative mRNA expression of CCNA2 and CDKN1B (p27) due to the treatment with the peptides. Cyclin E1, a protein encoded by the CCNE1 gene, accumulates at the S/G2 phase transition of the cell cycle and is subsequently degraded as the cell cycle advances into the S phase. CCNA2, on the other hand, associates with CDK2 during the S phase, with p53 playing a role in regulating CDK2 expression through the modulation of p21. With the decrease in CCNA2 and p53, perturbations in the S-to-G2 phase transition of the cell cycle become apparent, leading to the heightened expression of CCNE1 and CCND1 and reduced expression of CDKN1B (Figure 5).

Taken together, these outcomes strongly suggest that the treatment with N0820 and N0821 leads to the downregulation of CCNA2 and CDKN1B and the upregulation of CCNE1 and CCND1 in the DU145 cells. This, in turn, induces cell cycle arrest at the S/G2 phase transition.

## 4. Discussion

Trp-Arg dense-region peptides are renowned for their antibacterial properties. However, it is crucial to explore their potential as anticancer agents. These peptides, designed for cancer treatment, primarily target cell membranes, suggesting a low likelihood of resistance development to these anticancer drugs. In prostate cancer, the standard approach often involves surgical removal of the androgen-secreting gland. However, addressing androgen-independent prostate cancer remains challenging when hormone-based therapies are less effective. Consequently, there is a pressing need for comprehensive research on novel anticancer drugs.

The Trp-Arg dense-region peptide, derived from bee sources, was validated for its anti-prostate-cancer potential, primarily through the induction of apoptosis and cell cycle arrest. The MTT assay substantiates the peptides’ capability to trigger cell death, and this effect is consistently observed within a 3D spheroid environment.

Notably, the Western blot results indicate a declining trend in the p53 and p-p53 levels, leading to an upregulation in SERCA and MCU expression. This upregulation enables the peptide to function as an ion channel, facilitating the transfer of elevated Ca^2+^ from the cytoplasm to the mitochondria. These outcomes were anticipated to trigger apoptosis [43], a hypothesis substantiated by the Annexin V/PI staining results, which unequivocally confirm the effective induction of apoptosis by both peptides. The observed downregulation of CCNA2 and upregulation of CCNE1 strongly suggest the initiation of S/G2 cell cycle arrest. Of particular interest, N0821, a modified derivative of N0820 with a leading tyrosine unit, exhibited more pronounced alterations in all experiments except the MTT assay. It can be predicted that the first tyrosine unit in N0821 has hydrophilic properties and is therefore better embedded into the membrane, but additional research in this direction appears warranted.

## 5. Conclusions

In summary, the findings strongly support the antiproliferation potential of the Trp-Arg dense-region peptide in the therapy of androgen-independent prostate cancer. We believe that it is essential to study the mechanism by which Trp-Arg dense-region peptides exert antiproliferation efficacy and their effect on cell membranes and mitochondrial membranes.

Moreover, in elucidating the heightened antiproliferation potency of N0821, it is imperative to investigate the augmentation of its efficacy through the incorporation of specific amino acids at the N-terminal or C-terminal of the peptide [44].

This peptide holds promise as a substantial anticancer agent for the treatment of androgen-independent prostate cancer, which poses challenges for hormone-based therapies. There is a possibility of synergistic effects when combined with other anticancer medications, warranting further investigation in this regard.

## Figures and Tables

**Table 1:** Bee protein sequences used for peptide prediction.

Bee Species	Trp-Arg Rich Region in Bee Sequence
*Apis cerana*	RRSWWCCCWYWWWWRWWRKILGRARWKCRR
*Apis dorsata*	RRSWWCCCWYWWWWRWWRKIFGRARWKCRR
*Apis mellifera*	RRSWWCCCWYWWWWRWWRKMFGRARWKCRR
*Apis florea*	RRSWWCCCWYWWWWRWWRKMFGRARWKCRR

**Table 2:** The traits of bee species.

Bee Species	Species Characteristics
*Apis cerana*	The species referred to as the Oriental honeybee is predominantly distributed throughout East Asia. Characterized by their relatively smaller size, they predominantly inhabit forested regions.
*Apis dorsata*	The species commonly referred to as the giant honeybee is primarily found in Asia and the South Pacific region. These bees are known for their large size and gregarious nature, forming expansive colonies. They typically construct their hives on tree branches, often in large clusters.
*Apis mellifera*	The Western honeybee exhibits a global distribution and is recognized as one of the most widely studied species of honey bee. With variations in size, they demonstrate adaptability to diverse environmental conditions and are capable of reproductive success across various habitats.
*Apis florea*	The species referred to as the Little Oriental Bee inhabits regions of East and South Asia. It is distinguished by its diminutive size and primarily forages nectar from small flowers.

**Table 3:** Peptide structure prediction using PEP-FOLD4.

Name	Peptide Sequence
N0820	WWWWRWWRKI
N0821	YWWWWRWWRKI
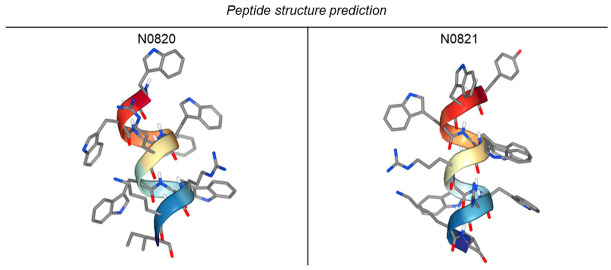	

**Table 4:** Primer list used in qRT-PCR.

Gene	Primer Sequence (5′ to 3′)	Annealing Temp. (°C)	References
*GAPDH*	F: GTGAAGGTCGGAGTCAACG R: TGAGGTCAATGAAGGGGTC	57.1 55.3	[35]
*CDKN2A* *(p14)*	F: GGTTCTTGGTGACCCTC R: CTAGACGCTGGCTCCTCAGT	59.5 58.4	[This study]
*CDKN1B* *(p27)*	F: AAGGTTTGGAGAGCGGCTG R: GATCAAATGGACTGGCGAGC	53.0 52.8	[This study]
*CCNA2* *(Cyclin A2)*	F: AGTAAACAGCCTGCGTTCACC R: GAGGGACCAATGGTTTTCTGG	59.1 57.1	[36]
*CCNB1* *(Cyclin B1)*	F: TAAGGCGAAGATCAACATGG R: GCTTCCTTCTTCATAGGCAT	53.0 52.8	[37]
*CCND1* *(Cyclin D1)*	F: CTGTGCTGCGAAGTGGAAACC R: GACGATCTTCCGCATGGAC	60.6 57.3	[25]
*CCNE1* *(Cyclin E1)*	F: ACACCATGAAGGAGGACG R: CACAGACTGCATTATTGTCCC	55.6 54.5	[25]

## Data Availability

All the data generated and analyzed during this study are included in this published article.
